# Efficacy and safety of enzyme replacement therapy with BMN 110 (elosulfase alfa) for Morquio A syndrome (mucopolysaccharidosis IVA): a phase 3 randomised placebo-controlled study

**DOI:** 10.1007/s10545-014-9715-6

**Published:** 2014-05-09

**Authors:** Christian J. Hendriksz, Barbara Burton, Thomas R. Fleming, Paul Harmatz, Derralynn Hughes, Simon A. Jones, Shuan-Pei Lin, Eugen Mengel, Maurizio Scarpa, Vassili Valayannopoulos, Roberto Giugliani, STRIVE Investigators, Peter Slasor, Debra Lounsbury, Wolfgang Dummer

**Affiliations:** 1 Birmingham Children’s Hospital NHS Foundation Trust, Birmingham, UK; 2 Ann and Robert H. Lurie Children’s Hospital and Northwestern University Feinberg School of Medicine, Chicago, IL USA; 3 Department of Biostatistics, University of Washington, Seattle, WA USA; 4 Children’s Hospital and Research Center Oakland, Oakland, CA USA; 5 Royal Free and University College Medical School, London, UK; 6 Manchester Centre for Genomic Medicine, St Mary’s Hospital, CMFT, MAHSC, University of Manchester, Manchester, UK; 7 Mackay Memorial Hospital and Mackay Medical College, Taipei, Taiwan; 8 Villa Metabolica, Centre for Pediatric and Adolescent Medicine, MC University of Mainz, Mainz, Germany; 9 Department of Pediatrics, University of Padova, Padova, Italy; 10 Hôpital Necker-Enfants Malades and IMAGINE Institute, Paris, France; 11 Medical Genetics Service/HCPA, Department of Genetics/UFRGS and INAGEMP, Porto Alegre, Brazil; 12 BioMarin Pharmaceutical Inc., Novato, CA USA; 13 Manchester Academic Health Science Centre, The Mark Holland Metabolic Unit, Salford Royal Foundation NHS Trust, Ladywell NW2- 2nd Floor Room 107, Salford, Manchester, M6 8HD UK

## Abstract

**Objective:**

To assess the efficacy and safety of enzyme replacement therapy (ERT) with BMN 110 (elosulfase alfa) in patients with Morquio A syndrome (mucopolysaccharidosis IVA).

**Methods:**

Patients with Morquio A aged ≥5 years (*N* = 176) were randomised (1:1:1) to receive elosulfase alfa 2.0 mg/kg/every other week (qow), elosulfase alfa 2.0 mg/kg/week (weekly) or placebo for 24 weeks in this phase 3, double-blind, randomised study. The primary efficacy measure was 6-min walk test (6MWT) distance. Secondary efficacy measures were 3-min stair climb test (3MSCT) followed by change in urine keratan sulfate (KS). Various exploratory measures included respiratory function tests. Patient safety was also evaluated.

**Results:**

At week 24, the estimated mean effect on the 6MWT versus placebo was 22.5 m (95 % CI 4.0, 40.9; *P* = 0.017) for weekly and 0.5 m (95 % CI −17.8, 18.9; *P* = 0.954) for qow. The estimated mean effect on 3MSCT was 1.1 stairs/min (95 % CI −2.1, 4.4; *P* = 0.494) for weekly and −0.5 stairs/min (95 % CI −3.7, 2.8; *P* = 0.778) for qow. Normalised urine KS was reduced at 24 weeks in both regimens. In the weekly dose group, 22.4 % of patients had adverse events leading to an infusion interruption/discontinuation requiring medical intervention (only 1.3 % of all infusions in this group) over 6 months. No adverse events led to permanent treatment discontinuation.

**Conclusions:**

Elosulfase alfa improved endurance as measured by the 6MWT in the weekly but not qow dose group, did not improve endurance on the 3MSCT, reduced urine KS, and had an acceptable safety profile.

**Electronic supplementary material:**

The online version of this article (doi:10.1007/s10545-014-9715-6) contains supplementary material, which is available to authorized users.

## Introduction

Morquio A syndrome (mucopolysaccharidosis [MPS] IVA; OMIM 253000) is a rare autosomal recessive lysosomal storage disorder caused by a deficiency in the enzyme *N*-acetylgalactosamine-6-sulfatase (GALNS; EC 3.1.6.4). This enzyme deficiency leads to an impaired degradation of the glycosaminoglycans (GAGs) keratan sulfate (KS) and chondroitin-6-sulfate (C6S) (Neufeld and Muenzer 2001). The incidence of Morquio A syndrome ranges from 1 in 640,000 live births in Western Australia to 1 in 76,000 live births in Northern Ireland (Nelson 1997; Nelson et al 2003).

Patients with Morquio A appear healthy at birth, but progressively develop a variety of clinical manifestations associated with the excessive GAG storage in tissues and organs and disruption in cellular processes (Yasuda et al 2013). The disease progression rate and the severity of clinical manifestations vary between patients. Typical clinical manifestations of Morquio A patients are profound skeletal dysplasia including dwarfism with short trunk and neck, spinal abnormalities, pectus carinatum, genu valgum, hip dysplasia, joint hypermobility and instability due to ligamentous laxity and bone deformities at the joint (Harmatz et al 2013; Neufeld and Muenzer 2001). In the longitudinal, prospective Morquio A Clinical Assessment Program (MorCAP) study including data from 325 Morquio A patients, over 90 % of patients showed skeletal dysplasia (Harmatz et al 2013). Other characteristic manifestations of the disease include dental abnormalities, spinal cord compression and myelomalacia, breathing difficulties due to airway obstruction and/or restrictive pulmonary disease, cardiac valve disease, impaired vision (e.g. corneal clouding, glaucoma), hearing loss and hepatosplenomegaly (Harmatz et al 2013; Hendriksz et al 2013; Neufeld and Muenzer 2001). Death commonly occurs due to cardiorespiratory or neurological complications (Harmatz et al 2013). Patients with a rapidly progressing phenotype generally die in their second or third decade of life (Harmatz et al 2013; Montaño et al 2007). Slowly progressing patients generally show less severe skeletal manifestations than those with a rapidly progressing course, but typical physical symptoms appear later in life and patients rarely survive beyond the sixth decade of life (Harmatz et al 2013).

Until recently, there was no approved treatment for Morquio A syndrome other than supportive care. Enzyme replacement therapy (ERT) with recombinant human GALNS (rhGALNS, elosulfase alfa, BMN 110, Vimizim®) represents a new treatment option that was approved in the US by the Food and Drug Administration in February 2014. Elosulfase alfa is expected to increase KS and C6S catabolism and reduce the KS and C6S accumulation contributing to the clinical manifestations.

Elosulfase alfa is produced in a genetically engineered Chinese Hamster Ovary (CHO) mutant cell line that expresses the cDNA encoding for the full human GALNS protein. It is identical to the naturally occurring human lysosomal enzyme in terms of the amino acid sequence and N‑linked glycosylation sites. rhGALNS is a soluble dimeric protein and each monomer contains 496 amino acids (after cleavage of the signal peptide) with a calculated isotope average molecular mass of 55 kDa for the peptide chain. The nonclinical pharmacology, pharmacokinetics and toxicology of elosulfase alfa have been evaluated in several in vitro and in vivo animal studies (Dvorak-Ewell et al 2010 and unpublished results), showing a toxicological and safety profile consistent with that observed in the nonclinical programs of other ERTs. The combined nonclinical data supported the clinical use of elosulfase alfa at the proposed dose and interval of 2 mg/kg/week via 4 h intravenous (iv) infusions for Morquio A patients. Further insights about the clinical efficacy and safety of elosulfase alfa were previously provided in a multicentre, phase 1/2, open-label, dose-escalation study including 20 Morquio A patients aged 5–18 years. Measures of 6-min walk test [6MWT], 3-min stair climb test [3MSCT]) and respiratory function were generally improved with elosulfase alfa 1.0 mg/kg/week and 2.0 mg/kg/week doses. Mean improvements over baseline were sustained after approximately 2 years, although there was no long-term control, there were missing data, and effort-dependent endpoints were evaluated in an open-label setting (Hendriksz et al 2012). In addition, urinary KS levels decreased, with the lowest levels occurring during dosing with the 2.0 mg/kg/week regimen.

The present phase 3 study was designed to assess the efficacy and safety of two dosing regimens of elosulfase alfa infusions in comparison with placebo in Morquio A patients.

## Materials and methods

### Study design

This was a multinational, multicentre, double-blind, randomised, placebo-controlled, 24-week phase 3 study (#NCT01275066). The study was conducted by 34 principal investigators at 33 study centres in 17 countries. A schematic presentation of the study design is shown in Supportive online material 1. After screening, eligible patients completed baseline assessments and were randomised to receive infusions of elosulfase alfa 2.0 mg/kg/week, elosulfase alfa 2.0 mg/kg/every other week (qow) or placebo for a period of 24 weeks. Randomisation was stratified by screening 6MWT category (≤200 and >200 m) and age group (5–11, 12–18, ≥19 years old). Patients, investigators and site personnel were blinded to treatment assignment throughout the study and until the final analysis was complete. All patients completing the first 24 weeks of the study were eligible for enrolment in an extension study.

### Patient selection

Study participants ≥5 years of age had a clinical diagnosis of Morquio A syndrome based on clinical signs and symptoms of the disease and documented reduced fibroblast or leukocyte GALNS enzyme activity or genetic testing confirming the diagnosis. Participants were also required to have an average 6MWT distance ≥30 and ≤325 m during screening, and had to be willing to use an acceptable method of contraception during the study (if sexually active). Patients who had previous hematopoietic stem cell transplantation, previous treatment with elosulfase alfa, major surgery within 3 months of study entry or planned surgery during the 24-week study treatment period, were pregnant or breastfeeding at screening or planning to become pregnant (self or partner) during the study, were using any investigational product or medical device within 30 days before screening or during the study, or had concurrent morbidity that would interfere with study participation or safety (e.g. symptomatic cervical spine instability, clinically significant spinal cord compression, severe cardiac disease) or that could interfere with treatment compliance were excluded from the study.

The rationale for including limits for baseline 6MWT distance of 30 to 325 m was to identify patients most likely to show improvement, i.e. excluding severely walk impaired patients with disease that has progressed to a point where walk impairment may not be reversible, and excluding those with walk distance approaching the normal range to avoid the potential for a ceiling effect.

### Study drug administration

Patients were pre-treated (approximately 30 to 60 min before each study drug infusion) with an antihistamine (preferably non-sedating, such as cetirizine or loratadine). Mandatory pre-treatment with anti-histamines was instituted during the prior phase 1/2 study and all subsequent studies to reduce the risk of infusion-associated reactions (IARs), which are to be expected upon infusion of large amounts of protein in general and based on previous experience with other ERTs. A sedating antihistamine or premedication with additional agents, such as H2 blockers, leukotriene receptor antagonists, steroids and/or antipyretic medications could be administered to patients with known risk factors for IARs, such as a history of IARs or allergies. IARs were defined as any adverse events (AEs) (regardless of drug relationship) that occurred after infusion onset and within 1 day after infusion end. Elosulfase alfa (2.0 mg/kg) or placebo solution was diluted with saline to a total volume of up to 250 mL at room temperature and administered iv. Patients randomised to elosulfase alfa 2.0 mg/kg/qow received infusions of placebo on alternating weeks to mask active drug weeks. Each infusion was administered over a period of approximately 4 h. For a volume of 100 mL, infusion rates were 3 mL/h for the first 15 min, then 6 mL for 15 min, then 6 mL increases every 15 min until a final rate of 36 ml/h until completed. For a volume of 250 mL, infusion rates were 6 mL/h for the first 15 min, then 12 mL for 15 min, then 12 mL increases every 15 min until a finale rate of 72 mL/h until completed.

### Efficacy evaluation

The primary efficacy measure was the distance walked in a 6MWT, which provides a measure of endurance. The 6MWT was performed according to American Thoracic Society (ATS) Guidelines (American Thoracic Society 2002). Secondary efficacy variables were 3MSCT, another measure of endurance, followed hierarchically by normalised urine KS. The 3MSCT was performed as previously described (Harmatz et al 2005). Both the 6MWT and 3MSCT were conducted twice on separate days at screening, week 12, and week 24 and within 1 week of early withdrawal from the study. Patients who were physically unable to perform the tests were scored as zero meters or stairs/min. The walk test score for each visit week was the average of the two measurements conducted for each time point. Study site personnel were thoroughly trained and certified in order to minimise variability in the conduct of the 6MWT and 3MSCT. Urine KS and creatinine were measured by quantitative analysis of samples obtained from first morning voids on two separate days at baseline and a single morning void at weeks 2 and 4 and every 4 weeks thereafter, as well as within 1 week of early withdrawal. A selective, sensitive and precise LC-MS/MS assay was used to measure the KS-derived disaccharides (Martell et al 2011). Urine KS concentrations normalised to creatinine concentrations were calculated.

Tertiary measures included pulmonary function measures such as forced vital capacity (FVC), forced expiratory volume in 1 s (FEV_1_) and maximum voluntary ventilation (MVV) conducted according to ATS guidelines (American Thoracic Society 1995). MVV results were included in a composite efficacy measure comprised of the average of the 24-week changes from baseline in normalised 6MWT, 3MSCT and MVV components. Each of the 6MWT, 3MSCT and MVV results were normalised as a z-score, using the calculated mean and standard deviation of all non-missing baseline scores as the reference. The composite measure was based on an equal weighting of week 24 change from baseline of the three component z-scores.

A number of additional tests and measures were included for exploratory purposes such as anthropometric measures (standing height, growth rate, sitting height, length and weight), hearing (measured using audiometry), cardiac valve function on echocardiogram, corneal clouding (as assessed by physical examination) and radiographic examinations (of cervical and lumbar spine; for subjects ≤20 years, lower extremity radiographs). Biochemical markers of inflammation (tumor necrosis factor α [TNFα]) and bone and cartilage metabolism (C-terminal crosslinked C-telopeptide [CTX1] and type IIA collagen N-propeptide [PIIANP]) were also determined. Quality of life (QoL) was assessed using the MPS Health Assessment Questionnaire (HAQ) which was originally developed to assess the self-care and mobility skills of patients with MPS I, and is currently used in an international MPS I Registry: (https://www.lsdregistry.net/mpsiregistry/hcp/partic/mreg_hc_p_healthassess.asp).

### Safety evaluation

Safety was assessed by examining incidence, severity and relationship to study drug of treatment-emergent AEs reported during the study. In addition, IARs, laboratory results (haematology, blood chemistry, urinalysis, thyroid function), vital signs, electrocardiogram and echocardiogram at screening and week 24, physical examination, concomitant medications and immunogenicity were assessed. Immunogenicity tests were performed using standardized immunogenicity assays on blood samples collected prior to study drug infusion at baseline and at weeks 2, 4, 8, 12, 16, 20 and 24. The total antibody assay measured multiple anti-drug antibody isotypes in one assay. Immunogenicity tests included elosulfase alfa-specific total antibody (TAb), neutralizing antibody (Nab, that inhibit cellular receptor binding), elosulfase alfa-specific immunoglobulin (Ig) E; and total IgE (baseline only). When TAb was negative, NAb was not assessed. Subjects with a severe IAR or IAR requiring infusion cessation had an additional blood sample taken to assess total IgE, drug specific IgE levels, complement, and tryptase. Potential hypersensitivity AEs were identified by utilising the broad Anaphylactic Reaction algorithmic Standardized Medical Dictionary of Regulatory Activities (MedDRA) query (SMQ) (MedDRA, version 15.0, http://www.meddra.org/) and the broad Angioedema SMQ.

### Statistical methods

It was calculated that approximately 162 subjects (54 subjects in each group) were to be enrolled into the study to have over 90 % power to detect a difference of 40 m in mean change in the 6MWT distance between the elosulfase alfa groups and the placebo group, assuming that the common standard deviation is 65 m with an overall 0.05 two sided significance level with Hochberg method for multiplicity adjustment.

The modified intent-to-treat efficacy analyses included all subjects who were randomised to study treatment and received at least one dose of study drug. The primary efficacy endpoint was the change from baseline at week 24 in the 6MWT distance. As the primary analysis method, the change in 6MWT distance from baseline to week 24 was compared between the placebo group and the elosulfase alfa 2.0 mg/kg/week and the elosulfase alfa 2.0 mg/kg/qow-treated groups separately, using an analysis of covariance (ANCOVA) model with baseline 6MWT category (≤200 and >200 m) and age group (5–11, 12–18, ≥19 years) as the covariates. As a supportive analysis, a cumulative distribution function of change from baseline at week 24 in 6MWT was plotted by treatment group. Exploratory analyses were performed to obtain clues about whether treatment effects differed across the levels of several baseline covariates, including the screening 6MWT, age categories, sex, race and region. The secondary endpoints (change from baseline at week 24 in the number of stairs climbed per minute in the 3MSCT and in the normalised urine KS) and additional supportive endpoints were analysed similarly to the 6MWT. As an adjustment for multiplicity with the secondary endpoints, a step-down testing procedure was used. The results of the 3MSCT were tested first, and the urine KS results could only be declared significant if the 3MSCT showed a significant result due to the requirement of the pre-specified statistical analysis plan. For the primary and secondary endpoints, the Hochberg method for multiplicity adjustment was used for the two treatment comparisons with placebo (Hochberg 1988). There was only one subject with missing data points for 6MWT at 24 weeks. Multiple imputation was used in the primary analysis of primary, secondary and respiratory function test endpoints (Rubin 1987) when a measurement was not available. The imputation was based on the joint normal distribution for the endpoint at all scheduled assessments, baseline age and baseline 6MWT scores.

Safety analysis included all patients who received any study drug (either elosulfase alfa or placebo) throughout the study duration. The incidence rates for AEs were summarised by System Organ Class, Preferred Term, relationship to study drug and severity for each treatment group.

## Results

### Patient characteristics

Of the 204 patients who were screened, 177 were randomised. One randomised patient was not dosed and excluded from all analyses because the diagnosis of Morquio A syndrome was not confirmed. Of the 176 patients, 59 were randomised to placebo, 59 to elosulfase alfa 2.0 mg/kg/qow and 58 to elosulfase alfa 2.0 mg/kg/week; 175 patients completed the study. One patient in the elosulfase alfa 2.0 mg/kg/week population discontinued after the first infusion due to withdrawal of consent. The withdrawal was due to logistic difficulties for attending study visits and not because of safety concerns.

All treatment groups achieved high mean dosing compliance (range 96.8 to 99.2 %) and few efficacy data points were missing. Baseline demographics and clinical characteristics of the patients are listed in Supportive online material 2, showing a broad distribution of ages ranging from 5 to 57 years (median age 11.9 year), races and ethnicity and an equal representation between genders (54.2 % females). There were no meaningful imbalances between treatment groups at baseline in demographic and baseline characteristics. 6MWT distance ranged from 36 to 322 m.

The entry criteria for 6MWT and the mean baseline 6MWT distance of 210 m confirm that patients were significantly impaired in endurance/mobility. Patients showed a wide variation in their functional impairment and organ system involvement. Medical history findings were similarly distributed across treatment groups and reflected the serious morbidities that Morquio A patients experience (Supportive online material 3). At least one abnormal medical history finding was reported for ≥98.3 % of patients in each group, with knee deformity, corneal opacity and pectus carinatum being most common.

### Primary efficacy measure: 6MWT

**Fig. 1:**
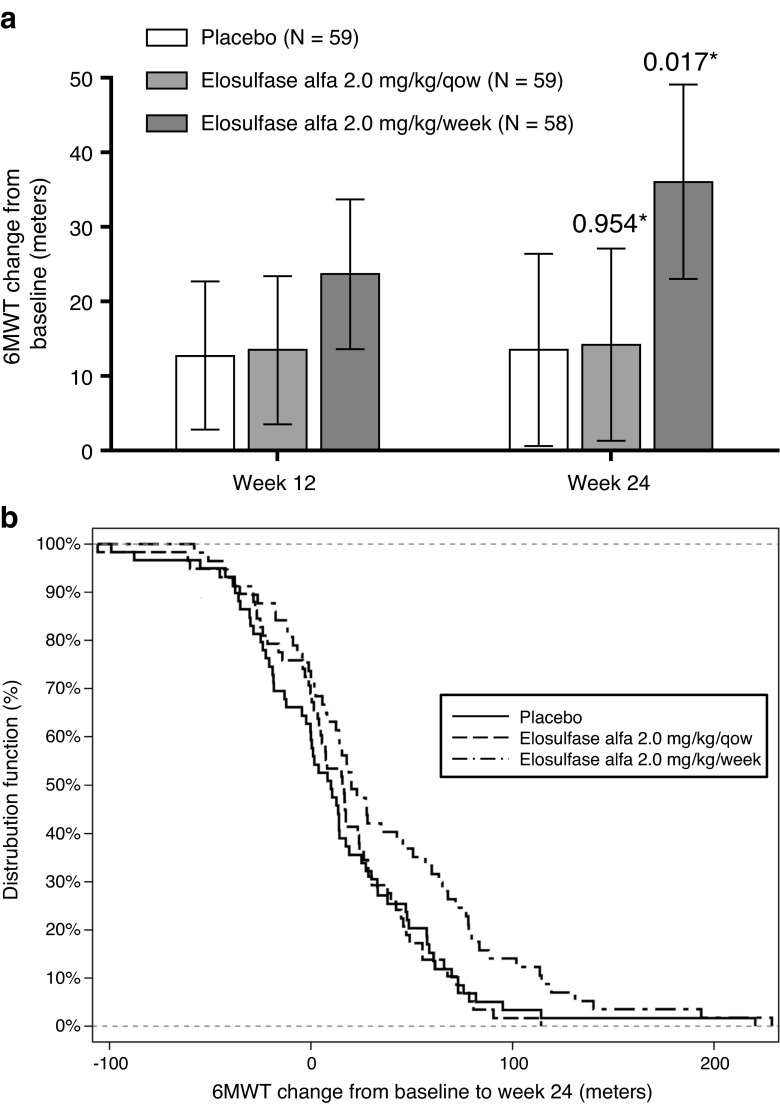
**a** Mean change from baseline over time in distance walked in a 6-min walk test (6MWT) (model-based repeated measures ANCOVA). *Error bars* represent 95 % CI of least squared mean change from baseline. *P-value vs. placebo. Qow: every other week. **b** Cumulative distribution plot of response in 6 min walk test (6MWT) distance (ITT population) based on observed data at week 24: for any selected walk test distance on the x-axis the proportion of subjects achieving the 6MWT distance is shown for each treatment group

The primary efficacy outcome at week 24 was evaluated using ANCOVA model based analysis (Table 1). The estimated mean effect on the 6MWT versus placebo was 22.5 m (95 % CI 4.0, 40.9; *P* = 0.017) for the elosulfase alfa 2.0 mg/kg/week regimen, and 0.5 m (95 % CI −17.8, 18.9; *P* = 0.954) for the elosulfase alfa 2.0 mg/kg/qow regimen. Accounting for the pre-specified adjustment for comparison of both regimens with placebo, the effect on the weekly regimen did meet the threshold for statistical significance (*P* = 0.017 < 0.025) for the primary end point of the trial. Figure 1a shows the least-square mean change from baseline over time for the placebo and treatment groups. Elosulfase alfa 2.0 mg/kg/week improved walking distance, relative to placebo, by 11 m at week 12 and by 23 m at week 24 (Supportive online material 4). The estimated cumulative distribution function in the placebo and elosulfase alfa treatment groups is provided in Fig. 1b.

**Table 1:** The effect of elosulfase alfa on primary, secondary and tertiary efficacy endpoints (intent-to-treat population). Modelled treatment effect, defined as (change from baseline to week 24, elosulfase alfa)–(change from baseline to week 24, Placebo)

Modelled treatment effect^a^	2.0 mg/kg/qow vs. placebo (*N* = 59 vs. 59)	2.0 mg/kg/week vs. placebo (*N* = 58 vs. 59)
Primary outcome		
6MWT distance (meters change from BL)		
LS Mean difference (95 % CI)	0.5 (−17.8, 18.9)	22.5 (4.0, 40.9)
*P*-value^b^	0.954	0.017
Secondary outcome		
3MSCT (stairs/min change from BL)		
LS mean difference (95 % CI)	−0.5 (−3.7, 2.8)	1.1 (−2.1, 4.4)
*P*-value^c^	0.778	0.494
Normalised urine KS (% change from BL)		
LS mean difference (95 % CI)	−30.2 (−38.5, −22.0)	−40.7 (−49.0, −32.4)
Tertiary outcome		
MVV (% change from BL)		
LS mean difference (95 % CI)	3.4 (−9.9, 16.6)	10.3 (−1.8, 22.4)
6MWD/3MSC/MVV composite z-score (change from BL)		
LS mean difference (95 % C)	0.0 (−0.1, 0.2)	0.1 (−0.0, 0.3)
FVC (% change from BL)		
LS mean difference (95 % CI)	3.0 (−2.4, 8.3)	3.3 (−3.1, 9.6)
FEV_1_ (% change from BL)		
LS mean difference (95 % CI)	0.2 (−7.4, 7.9)	1.8 (−5.5, 9.2)
MPS HAQ		
Self care domain^d^ score (change from BL)		
LS mean difference (95 % CI)	−0.1 (−0.5, 0.3)	0.1 (−0.3, 0.5)
Caregiver assistance domain^d^ score (change from BL)		
LS mean difference (95 % CI)	−0.4 (−2.4, 1.6)	−0.9 (−2.8, 1.1)
Mobility domain^d^ score (change from BL)		
LS mean difference (95 % CI)	−0.3 (−0.8, 0.2)	−0.3 (−0.8, 0.3)
Anthropometric measures		
Standing height z-score^e^ (change from BL)		
LS mean difference (95%CI)	0.1 (−0.1, 0.3)	0.1 (−0.0, 0.3)
Growth rate z-score^e^ (change from BL)		
LS mean difference (95 % CI)	0.4 (−0.1, 0.9)	0.4 (−0.1, 0.9)

*3MSCT* 3-min stair climb test; *6MWT* 6-min walk test; *BL* baseline; *CI* confidence interval; *FEV1* forced expiratory volume in 1 s, *FVC* forced vital capacity; *HAQ* health assessment questionnaire; *KS* keratan sulfate; *LS mean* least squares mean; *MPS* mucopolysaccharidosis; *MVV* maximum voluntary ventilation; *qow* every other week

^a^Treatment effect defined as (change from baseline to week 24, elosulfase alfa)–(change from baseline to week 24, placebo)

^b^ *P*-value determined by *t*-test from ANCOVA model (primary endpoint analysis), adjusted for baseline covariates: age group and 6-min walk test (6MWT) category

^c^ *P*-value determined by *t*-test ANCOVA model adjusted for baseline covariates: age group, 6MWT category, and continuous 3-min stair climb test

^d^MPS HAQ domain scores range from 0 to 20; decreases in domain scores (negative values) indicate improvement

^e^The normalised standing height (scaled as a z-score) computed using CDC normals was explored in males ≤18 years and females ≤15 years; the combined historical and on study normalized standard height z-scores were used to compute growth rate change (scaled as z-score)

Tertiary variables that were analysed differently from those included in the table are discussed in the text

Sensitivity analyses for effects on 6MWT were consistent with the primary analysis. The sensitivity analyses included analysis of the first, second and best of the duplicate measurements performed on two separate days at each visit. Analyses omitting outliers and using alternative methods for handling missing observations including observed cases were also performed. There were very few missing observations. Subgroup analyses did not provide strong evidence of effect modification by any of the categories of age group, sex, race and geographic region (see Supportive online material 5). However, there was a trend for larger effects on 6MWT in patients with baseline 6MWT ≤ 200 m and for smaller effects in patients with baseline 6MWT > 200 m.

### Secondary and tertiary measures

On the 3MSCT (stairs/min), the elosulfase alfa 2.0 mg/kg/week group showed a small numerical increase and elosulfase alfa 2.0 mg/kg/qow group showed a small numerical decrease as compared to placebo, without statistical significance (Table 1, Supportive online material 6).

Given the hierarchical analysis of the secondary endpoints (with the 3MSCT tested first and not reaching statistical significance), the results for the remaining secondary and tertiary endpoints are presented in a descriptive manner in Table 1. *P*-values of these endpoints are provided in Supportive online material 7.

**Fig. 2:**
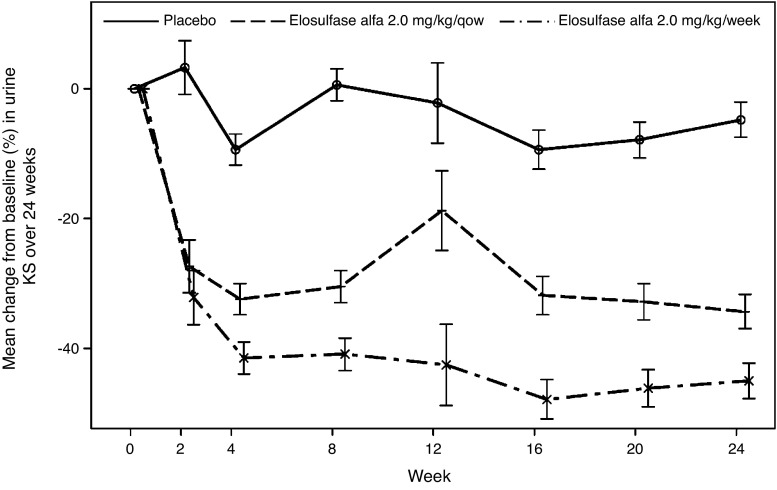
Mean percent change from baseline over time in normalised urine keratan sulfate (KS) (model-based repeated measures ANCOVA). Error bars represent standard error of least squared mean change from baseline. No *P*-values are given as a step-down testing procedure was used as an adjustment for multiplicity with the secondary endpoints; this testing procedure implied that the urine KS results could only be declared significant if the 3-min stair climb test showed a significant result. qow: every other week

Treatment with elosulfase alfa led to an estimated reduction in urine KS in both treatment arms (Fig. 2), specifically being −30.2 % for the qow dosing regimen and −40.7 % for the weekly dosing regimen (Table 1) when compared with placebo at week 24.

For the tertiary variable MVV, numerical improvement favouring the elosulfase alfa regimens were seen when compared to placebo. Regarding the composite score combining 6MWT, 3MSCT and MVV z-scores at week 24, the estimated treatment effect at week 24 versus placebo was 0.1 (95 % CI −0.0, 0.3) for elosulfase alfa 2.0 mg/kg/week and 0.0 (95 % CI −0.1, 0.2) for elosulfase alfa 2.0 mg/kg/qow (Table 1).

For the other exploratory measures, treatment with elosulfase alfa (both dosing regimens) led to small to modest differences that were in the positive direction for the majority, though with wide confidence intervals. These measures include FVC, FEV_1_, QoL (Caregiver Assistance Domain and Mobility Domain scores of the MPS HAQ), standing height and growth rate z-scores in males ≤ 18 years and females ≤ 15 years (Table 1). Differences in audiometry, echocardiogram, corneal clouding, or lower extremity bone length were small. Given the limited duration of the trial and the natural history of Morquio A syndrome, statistically precise estimates of effects on these measures were unlikely to be achieved. Elosulfase alfa treatment also was not associated with a reduction in the blood inflammatory marker TNFα or changes in PIIANP or CTX1 levels, which are products of increased cartilage and bone metabolism.

### Safety

Table 2 summarises treatment-emergent AEs and serious adverse events (SAEs) reported for the different treatment groups. The most frequent AEs were mild to moderate IARs such as vomiting, pyrexia and headache, which were generally manageable with symptomatic treatment and/or infusion rate modification. IARs occurred in 91.5 %, 94.9 % and 89.7 % of patients treated with placebo, elosulfase alfa 2.0 mg/kg/qow and elosulfase alfa 2.0 mg/kg/week, respectively. All patients who experienced an IAR received and tolerated subsequent infusions. Hypersensitivity AEs occurred in 11.9 %, 27.1 % and 20.7 % of patients treated with placebo, elosulfase alfa 2.0 mg/kg/qow and elosulfase alfa 2.0 mg/kg/week, respectively, and were mostly mild or moderate in severity. While 22.4 % of the 58 patients receiving elosulfase alfa 2.0 mg/kg/week group had at least 1 AE over 6 months leading to an infusion interruption/discontinuation requiring medical attention, none withdrew or discontinued treatment due to an AE. Also, of the 1345 total infusions administered over 6 months to this treatment cohort, only 17 (1.3 %) were interrupted or discontinued (14 and 3, respectively) and also required medical intervention.

**Table 2:** Overall summary of treatment-emergent adverse events (AEs) and overview of serious adverse events (SAEs). Number (%) of patients in safety population

	Placebo (*n* = 59)	Elosulfase alfa 2.0 mg/kg/qow (*n* = 59)	Elosulfase alfa 2.0 mg/kg/wee (*n* = 58)
Any treatment-emergent AE	57 (96.6 %)	59 (100.0 %)	56 (96.6 %)
Mild	36 (61.0 %)	33 (55.9 %)	28 (48.3 %)
Moderate	20 (33.9 %)	23 (39.0 %)	26 (44.8 %)
Severe	1 (1.7 %)	3 (5.1 %)	2 (3.4 %)
Any study drug-related AE^a^	36 (61.0 %)	42 (71.2 %)	42 (72.4 %)
Mild	32 (54.2 %)	27 (45.8 %)	24 (41.4 %)
Moderate	4 (6.8 %)	14 (23.7 %)	16 (27.6 %)
Severe	0	1 (1.7 %)	2 (3.4 %)
Any AE leading to dose interruption/discontinuation requiring medical attention	0	9 (15.3 %)	13 (22.4 %)
Any AE leading to permanent study drug discontinuation	0	0	0
SAEs			
At least 1 reported SAE	2 (3.4 %)	4 (6.8 %)	9 (15.5 %)
Pneumonia	0	0	2 (3.4 %)
Hypersensitivity	0	0	1 (1.7 %)
Infusion site pain	0	0	1 (1.7 %)
Lower respiratory tract infection	0	0	1 (1.7 %)
Otitis media	0	1 (1.7 %)	1 (1.7 %)
Urticaria	0	0	1 (1.7 %)
Viral upper respiratory tract infection	0	0	1 (1.7 %)
Vomiting	0	0	1 (1.7 %)
Anaphylactic reaction	0	1 (1.7 %)	0
Cervical cord compression	1 (1.7 %)	0	0
Deafness	1 (1.7 %)	0	0
Dengue fever	0	1 (1.7 %)	0
Suture removal	0	1 (1.7 %)	0

^a^A drug-related AE was classified by investigator as possibly or probably related to study drug

AEs and SAEs coded by MedDRA version 15.0; maximum severity is summarised by subject

Severity categories: mild, no limitation of usual activities; moderate, some limitation of usual activities; severe, inability to carry out usual activities

The percent of subjects with SAEs was 3.4 % for placebo, 6.8 % for elosulfase alfa qow and 15.5 % for elosulfase alfa once weekly arm. The majority of SAEs in the weekly dose group appeared to be either infusion-related, procedure-related or Morquio A disease related. Three elosulfase alfa-treated patients had SAEs that were considered by the investigator to be related to the study drug: (1) one anaphylactic reaction that resolved the same day with treatment and reduction of infusion rate (qow group), (2) one hypersensitivity reaction that resolved within 24 h with symptomatic medical treatment and infusion discontinuation (weekly group) and (3) one case of severe vomiting that resolved the same day without medication (weekly group). None of these SAEs led to study discontinuation. No deaths occurred on study.

All patients treated with elosulfase alfa developed anti-drug antibodies and the majority developed antibodies capable of interfering with CI-M6PR binding in vitro (NAb). Despite the high incidence of anti-elosulfase alfa antibodies, decreases in urine KS and improvements in 6MWT were observed in treated subjects. No correlations were detected between higher TAb titers or NAb positivity and reduced 6MWT results or increased incidence of hypersensitivity AEs. Less than 10 % of subjects tested positive for drug-specific IgE during the study. Drug-specific IgE positivity was not associated with the occurrence of anaphylaxis, other hypersensitivity AEs and/or treatment withdrawal.

## Discussion

Morquio A syndrome is a progressive and serious disorder causing extensive morbidity and early mortality. The clinical presentation of Morquio A is very heterogeneous, resulting in a wide variety of specific phenotypes (Harmatz et al 2013; Montaño et al 2007). The disease represents a significant unmet medical need because there are currently no available treatment options that specifically correct the biological cause of the condition.

Individual cases of hematopoietic stem cell transplantation (HSCT) in patients with Morquio A have been reported showing some clinical benefit, but no impact on skeletal manifestations (Tomatsu et al 2011; Algahim and Almassi 2013). Considering the limited experience and the high morbidity associated with HSCT, this therapy is currently not recommended for Morquio A syndrome. The present study was designed to determine whether ERT with elosulfase alfa could be a therapy for Morquio A syndrome. ERT replaces the deficient GALNS enzyme by a functioning enzyme (rhGALNS or elosulfase alfa), thus repairing the mechanism for degrading keratan sulfate and chondroitin sulfate which is believed to lie at the basis of the disease manifestations. The dosing regimen of 2.0 mg/kg/week was selected on the basis of data from a phase 1/2 clinical study (Hendriksz et al 2012) evaluating dose escalation which showed greatest reduction in urine KS at 2.0 mg/kg and on nonclinical and in vitro studies with elosulfase alfa (Dvorak-Ewell et al 2010). A more convenient 2.0 mg/kg/qow dosing regimen was also evaluated to determine whether patients could derive benefit (and a lower risk of AEs) with less frequent dosing and lower cumulative exposure.

While not a direct measure of the effect of treatment on how patients feel, function or survive, the 6MWT provides an indirect assessment of endurance and functionality (Fleming and Powers 2012). Due to its enhanced sensitivity to treatment effect, it has been used in a variety of patient populations, including those with moderate to severe heart and lung diseases, neurological diseases and musculoskeletal diseases (Butland et al 1982; McDonald et al 2010). The test was first used for registration purposes in patients with pulmonary hypertension (Barst et al 1996). The 6MWT (or 12 MWT as a variation) has been the primary outcome or component of a composite outcome in all three previously approved therapies for MPS disease (Harmatz et al 2006; Muenzer et al 2006; Wraith et al 2004). Although it remains unclear whether the improvement in MPS disease is related to change in muscle strength, joint function/pain, cardiac function or pulmonary function or some combination of these parameters, it has been the most useful marker of drug effect on physical function in this population. The very short stature, severe bone and joint abnormalities, muscle weakness and very limited lung capacity in the MPS population presents a challenge in finding a functional task that is within the capacity of patients to perform reliably, reproducibly and according to standardised guidelines. The 6MWT also has been a sensitive and rapidly responsive marker, which is essential for the short-term trials required for MPS ERT trials because of the invasiveness of placebo intervention (i.e. weekly iv infusions) and the high frequency of MPS disease-related complications and surgeries. The 6MWT has been shown to meet this challenge in three previous MPS disease phase 3 trials, in contrast to pulmonary function tests and a number of tertiary outcome variables such as joint range of motion, grip and pinch strength, coin pick-up, QoL assessments, growth and survival.

In this clinical trial in Morquio A patients, an improvement in 6MWT distance on elosulfase alfa 2.0 mg/kg/week over placebo began to emerge at 12 weeks and became more apparent at week 24, where the placebo adjusted mean increase in 6MWT distance was 22.5 m. While it is not possible to specifically determine which body system was responsible for improvement in 6MWT distance in each patient, it is likely that it was a multifactorial effect based on the pathophysiology of the condition. However, the difference in 6MWT distance with placebo at 24 weeks was only 0.5 m in the elosulfase alfa 2.0 mg/kg/qow cohort. Accounting for the pre-specified adjustment for comparison of both regimens with placebo, the effect on the weekly regimen did meet the threshold for statistical significance. The trial was designed to detect a placebo-adjusted improvement of 40 m. The reason the results were not robustly positive is that the estimated effect size was smaller.

There has not been a rigorous anchor-based assessment of the minimally clinically important difference (MCID) for 6MWT in Morquio A or other MPS diseases. If such an assessment could be developed, it would provide valuable insights into the clinical relevance of a placebo-adjusted difference of 22.5 m at 24 weeks in Morquio A syndrome, which is characterised by progressive debilitation, frequently leading to immobility and the need for life-long assistance with activities of daily living (Harmatz et al 2013; Montaño et al 2007). The mean screening 6MWT distance of the study population of around 210 m is markedly lower than that of a healthy pediatric population, ranging from 470 to 664 m (Lammers et al 2008; Li et al 2007), and significantly lower than the 370 m in the chronic obstructive pulmonary disease population, which provided a MCID of 54 m (Redelmeier et al 1997). A recent study in patients with Duchenne muscular dystrophy identified 5.9 m as the MCID for a baseline walk distance of 150 m, suggesting that smaller increases in 6MWT distance may result in meaningful changes in QoL measurements (Henricson et al 2013). The 22 m difference/improvement in the present study should fit in the spectrum between these two disease populations.

In the 3MSCT, both weekly and qow dosing groups did not show improvement over placebo by week 24. It is unknown why the 3MSCT results did not parallel the primary endpoint data. 3MSCT was included as a secondary endpoint based on its use in previous ERT studies in MPS VI patients (Harmatz et al 2005; Harmatz et al 2006; Harmatz et al 2008) and on results seen in the phase 1/2 clinical trial of elosulfase alfa in Morquio A patients (Hendriksz et al 2012). However the test may be more variable and less suitable for Morquio A patients, who tend to show more severe skeletal dysplasia and joint abnormalities and therefore have more ambulation difficulties (walking aid and wheelchair use) than the patients included in these MPS VI studies (Aslam et al 2013; Harmatz et al 2013; Hendriksz et al 2013; Montaño et al 2007). Also, it may be that 24 weeks is not a long enough observation period to detect a difference versus placebo, or that elosulfase alfa does not provide substantial benefits on the 3MSCT in this disease setting.

Urine KS is considered a pharmacodynamic biomarker for Morquio A syndrome, with higher levels being related to more severe clinical impairment (Harmatz et al 2013; Dũng et al 2013). Urine C6S was intentionally not measured as it accumulates to a lesser extent than does KS as it presumably has an alternative route for catabolism in addition to GALNS (Neufeld and Muenzer 2001). While there was an observed decline in urine KS on the elosulfase alfa regimens, it is noteworthy that the relative effects of the two elosulfase alfa regimens on the urine KS biomarker did not accurately predict their estimated relative effects on the 6MWT at 24 weeks. This could mean that either a correlation does not exist, that the primary clinical outcome measure did not detect such a correlation with the present study sample size, or that a threshold effect occurs for the endurance responses. More likely, the urine KS is highly sensitive to dose changes not seen in an endurance test impacted by multiple factors (i.e. surgeries, neurologic injury, joint abnormalities). In our study, there was a transient increase in urine KS at week 12 in the elosulfase alfa 2.0 mg/kg/qow group. The reason for this remains unclear.

The analyses of tertiary variables showed numerical improvements over placebo at 24 weeks in the elosulfase alfa arms for the majority of measures. However, for all single measures, including MVV, FVC, FEV_1_, growth rate, standing height and all MPS HAQ domains, the sizes of the estimated effects were modest. The group composite efficacy measure, comprising 6MWT, 3MSCT, and MVV measurements, reflected the positive trends from the 6MWT and MVV endpoints but, due to small effects on 3MSCT, failed to achieve significance.

Treatment compliance was high and almost all patients completed the study without treatment discontinuation. The safety profile of elosulfase alfa was generally similar to that of other ERTs (e.g. MPS I, II and VI) (Clarke et al 2009; Harmatz et al 2008; Muenzer et al 2006; Muenzer et al 2011). The most common side effects of elosulfase alfa were IARs, which were self-limiting and manageable with symptomatic treatment. The high rate of IARs was not unexpected for treatments with recombinant protein. Infusion reactions requiring dose interruption/discontinuation and also requiring medical attention were observed in 22.4 % of patients, but in only 1.3 % of all infusions given to the weekly dose group over the 24 weeks of the study. None of these IARs led to permanent study drug discontinuation. The percent of subjects with SAEs was approximately four-fold higher on the once weekly regimen than on placebo. A majority of the SAEs in the weekly dose group were either infusion-related, surgical procedure-related, or disease-related.

All patients receiving elosulfase alfa developed anti-elosulfase alfa antibodies. Although it is not possible to determine whether this had an effect on the magnitude of the efficacy responses or on the incidence of hypersensitivity AEs, it is of interest that neither total antibody titres nor NAb positivity were associated with hypersensitivity AEs.

The present study is the largest placebo-controlled trial conducted to date in MPS patients and has generated a reasonable safety database for this ultra-rare disease. The impact of elosulfase alfa on 6MWT distance and urine KS levels shows that the therapy indeed has the potential to correct the biological cause of Morquio A syndrome. However, the progressive nature of the disease warrants more, longer-term studies to assess how the therapy will influence the disease course in the long term, especially when ERT can be started before severe manifestations have developed.

The study population of the present study can be considered representative of the general Morquio A population. However, as the study did not include patients unable to walk or with near-normal endurance in a 6MWT during screening and no patients below 5 years of age, no conclusions can currently be made regarding the impact of elosulfase alfa on 6MWT distance in these patients. Other clinical trials including these patient populations are currently ongoing. Nevertheless, the patients in this study exhibited a wide spectrum of age, disease severity and clinical manifestations, with mean 6MWT, 3MSCT and pulmonary function data being comparable to those reported in the MorCAP study (Harmatz et al 2013). The very complete, robust and high quality data collected during the study and the consistency of duplicate test results in 6MWT (intraclass correlation coefficient [ICC] range 0.868-0.938 by visit and regimen) and 3MSCT (ICC range 0.881-0.968) assessments enhance the validity of the findings. The efforts made to maximise data quality resulted in a very high dosing compliance, with only 0.6-1.8 % of planned infusions missed, and a very low number of missing efficacy data points. Only two week 24 6MWT assessments were missed.

## Conclusions

In this double-blind, randomised, placebo-controlled pivotal phase 3 study in Morquio A patients, the pre-specified primary efficacy measure of 6MWT distance at 24 weeks improved in the elosulfase alfa 2.0 mg/kg weekly, but not the qow dose group. Both elosulfase alfa regimens did not improve endurance in the 3MSCT, but did lead to a reduction in urine KS. These regimens were shown to provide generally safe ERT for patients with Morquio A syndrome. Elosulfase alfa may present a new treatment for patients with Morquio A who have currently no medical care option other than symptomatic therapy of disease complications.

## Electronic supplementary material

Below is the link to the electronic supplementary material.

Supportive online material 1 (PDF 4 kb)

Supportive online material 2 (PDF 26 kb)

Supportive online material 3 (PDF 16 kb)

Supportive online material 4 (PDF 11 kb)

Supportive online material 5 (PDF 29 kb)

Supportive online material 6 (PDF 21 kb)

Supportive online material 7 (PDF 19 kb)
