# Supplementary figures and images for: Efficacy and safety of enzyme replacement therapy with BMN 110 (elosulfase alfa) for Morquio A syndrome (mucopolysaccharidosis IVA): a phase 3 randomised placebo-controlled study
Source: J Inherit Metab Dis. 2014 May 9;37(6):979–90. doi: 10.1007/s10545-014-9715-6 (PMC4206772; doi:10.1007/s10545-014-9715-6)

**Supportive online material 1:** Schematic representation of the study design

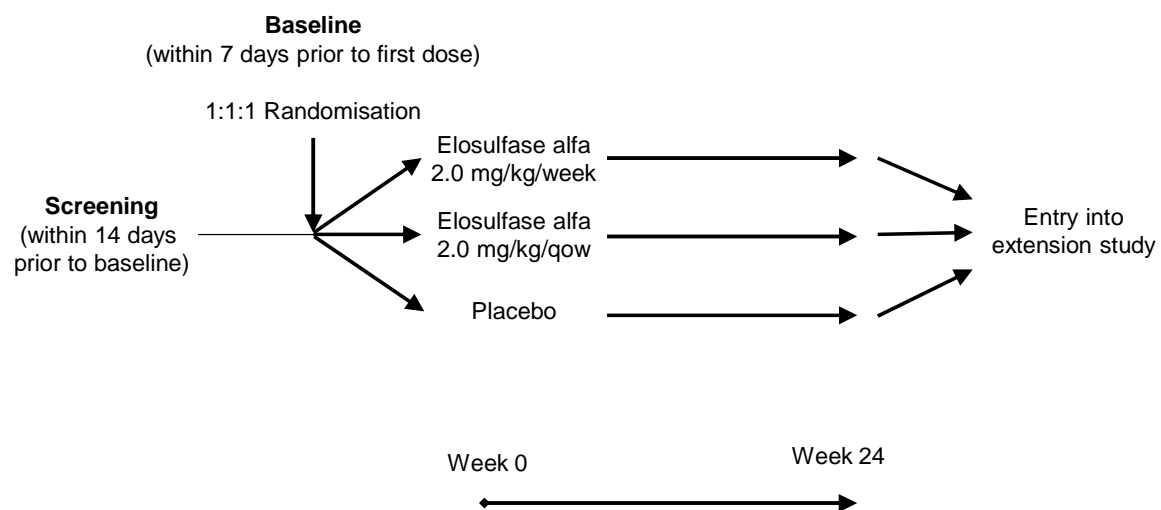

Supplement: Supplementary file 1 — (PDF 4 kb) [file 10545_2014_9715_MOESM1_ESM.pdf]
