# Supplementary material for: Efficacy and safety of enzyme replacement therapy with BMN 110 (elosulfase alfa) for Morquio A syndrome (mucopolysaccharidosis IVA): a phase 3 randomised placebo-controlled study
Source: J Inherit Metab Dis. 2014 May 9;37(6):979–90. doi: 10.1007/s10545-014-9715-6 (PMC4206772; doi:10.1007/s10545-014-9715-6)
Supplement: Supplementary file 2 — (PDF 26 kb) [file 10545_2014_9715_MOESM2_ESM.pdf]

Supportive online matrial 2: Baseline demographics and clinical characteristics

(Intent-to-Treat Population)

	Placebo	Elosulfase alfa 2.0 mg/kg/qow	Elosulfase alfa 2.0 mg/kg/week
**Baseline demographics**			
N	59	59	58
Age at Enrolment (years)			
Mean (SD)	15.0 (11.30)	15.3 (10.79)	13.1 (8.10)
Median (range)	11.9 (5-57)	12.0 (5-49)	11.1 (5-42)
Min - Max	5 - 57	5 - 49	5 - 42
Age Group (years)[a]			
5 - 11	30 (50.8%)	31 (52.5%)	32 (55.2%)
12 - 18	15 (25.4%)	16 (27.1%)	16 (27.6%)
≥ 19	14 (23.7%)	12 (20.3%)	10 (17.2%)
Sex			
Female	32 (54.2%)	25 (42.4%)	32 (55.2%)
Male	27 (45.8%)	34 (57.6%)	26 (44.8%)
Race			
Asian	11 (18.6%)	15 (25.4%)	14 (24.1%)
Black or African American	0	2 (3.4%)	2 (3.4%)
White	44 (74.6%)	35 (59.3%)	36 (62.1%)
Other	4 (6.8%)	7 (11.9%)	6 (10.3%)
Ethnicity			
Hispanic or Latino	13 (22.0%)	16 (27.1%)	9 (15.5%)
Other	46 (78.0%)	43 (72.9%)	49 (84.5%)
**Baseline clinical characteristics**			
6MWT (meters)			
N	59	59	58
Mean (SD)	211.9 (69.9)	205.7 (81.2)	203.9 (76.3)
Median (range)	228.9 (36-312)	218.0 (47-320)	216.5 (42-322)
Walking aids used			
N	11 (18.6%)	16 (27.1%)	9 (15.5%)
3MSCT (stairs/minute)			
N	59	59	58
Mean (SD)	30.0 (14.1)	27.1 (15.8)	29.6 (16.4)
Median (range)	30.8 (0-59)	25.5 (0-67)	30.5 (0-72)
Normalised urine KS[b] (µg/mg)			
N	58	59	58
Mean (SD)	25.7 (15.1)	28.6 (21.2)	26.9 (14.1)
Median (range)	26.7 (2-53)	27.4 (2-117)	24.1 (2-59)
MVV (L/min)			
N	51	53	52
Mean (SD)	34.8 (27.3)	32.8 (20.0)	28.3 (16.6)
Median (range)	27.0 (7.0-129.0)	26.8 (10.0-91.1)	25.0 (5.0-76.0)

	Placebo	Elosulfase alfa 2.0 mg/kg/qow	Elosulfase alfa 2.0 mg/kg/week
FVC (L)			
N	54	55	56
Mean (SD)	1.2 (0.9)	1.1 (0.7)	0.9 (0.5)
Median (range)	0.9 (0.3-5.0)	0.9 (0.3-3.0)	0.8 (0.3-3.0)
Standing height (cm)			
N	58	59	58
Mean (SD)	105.5	104.6	101.3
Median (range)	100.0 (86 – 165)	100.2 (81 – 147)	98.8 (83 – 141)

3MSCT: 3-minute stair climb test; 6MWT: 6-minute walk test; FVC: forced vital capacity; KS: keratan sulfate; MVV: maximum voluntary ventilation; qow: every other week; SD, standard deviation

[a] Stratification Factor; [b]normalized urine KS is calculated as urine keratan sulfate divided by urine creatinine
