# Supplementary material for: Efficacy and safety of enzyme replacement therapy with BMN 110 (elosulfase alfa) for Morquio A syndrome (mucopolysaccharidosis IVA): a phase 3 randomised placebo-controlled study
Source: J Inherit Metab Dis. 2014 May 9;37(6):979–90. doi: 10.1007/s10545-014-9715-6 (PMC4206772; doi:10.1007/s10545-014-9715-6)
Supplement: Supplementary file 3 — (PDF 16 kb) [file 10545_2014_9715_MOESM3_ESM.pdf]

Supportive online material 3: Medical history by treatment group: incidence ≥ 20% in any of the treatment groups by Preferred Term (Intent-to-Treat population)

**Preferred Term**	**Placebo (N = 59)**	**Elosulfase alfa 2.0 mg/kg/qow (N = 59)**	**Elosulfase alfa 2.0 mg/kg/week (N = 58)**
**Subjects with at least one reported medical history finding, n (%)**	58 (98.3%)	58 (98.3%)	58 (100.0%)
Corneal opacity, n (%)	30 (50.8%)	34 (57.6%)	30 (51.7%)
Knee deformity, n (%)	26 (44.1%)	33 (55.9%)	32 (55.2%)
Pectus carinatum, N (%)	23 (39.0%)	25 (42.4%)	27 (46.6%)
Tricuspid valve incompetence, n (%)	20 (33.9%)	15 (25.4%)	13 (22.4%)
Kyphosis, n (%)	18 (30.5%)	19 (32.2%)	17 (29.3%)
Body height below normal, n (%)	17 (28.8%)	23 (39.0%)	12 (20.7%)
Spinal fusion surgery, n (%)	15 (25.4%)	12 (20.3%)	12 (20.7%)
Dysmorphism, n (%)	13 (22.0%)	14 (23.7%)	9 (15.5%)
Hip dysplasia, n (%)	11 (18.6%)	11 (18.6%)	16 (27.6%)
Arthralgia, n (%)	11 (18.6%)	10 (16.9%)	14 (24.1%)
Ear tube insertion, n (%)	11 (18.6%)	12 (20.3%)	12 (20.7%)
Knee operation, n (%)	11 (18.6%)	10 (16.9%)	9 (15.5%)
Tooth malformation, n (%)	11 (18.6%)	13 (22.0%)	9 (15.5%)
Bone deformity, n (%)	9 (15.3%)	12 (20.3%)	13 (22.4%)

qow: every other week.

MedDRA version 15.0

Subjects who experienced more than one finding within a MedDRA category were counted once within that category.
