# Supplementary material for: Efficacy and safety of enzyme replacement therapy with BMN 110 (elosulfase alfa) for Morquio A syndrome (mucopolysaccharidosis IVA): a phase 3 randomised placebo-controlled study
Source: J Inherit Metab Dis. 2014 May 9;37(6):979–90. doi: 10.1007/s10545-014-9715-6 (PMC4206772; doi:10.1007/s10545-014-9715-6)
Supplement: Supplementary file 4 — (PDF 11 kb) [file 10545_2014_9715_MOESM4_ESM.pdf]

Supportive online material 4: Descriptive summary of 6-minute walk test (observed case). Intent-to-treat population

6-Minute Walk Test (meters)	Placebo (N = 59)	Elosulfase alfa 2.0 mg/kg/qow (N = 59)	Elosulfase alfa 2.0 mg/kg/week (N = 58)
**Baseline**			
n	59	59	58
Mean (SD)	211.9 (69.9)	205.7 (81.2)	203.9 (76.3)
Median	228.9	218.0	216.5
Min, Max	36.2, 312.2	47.1, 319.6	42.4, 321.5
**Week 12**			
n	59	59	58
Mean (SD)	224.6 (78.5)	219.1 (78.4)	227.6 (76.4)
Median	231.3	232.1	237.1
Min, Max	51.5, 431.5	54.7, 377.3	48.6, 350.7
**Week 24**			
n	59	58	57
Mean (SD)	225.4 (83.2)	220.5 (88.2)	243.3 (83.5)
Median	229.4	238.1	251.0
Min, Max	50.6, 501.0	44.1, 370.4	52.0, 399.9
**Week 12 - Change from Baseline**			
n	59	59	58
Mean (SD)	12.7 (35.8)	13.5 (38.4)	23.7 (42.2)
Median	11.4	13.6	21.4
Min, Max	-70.9, 137.0	-102.5, 106.8	-86.4, 171.0
**Week 24 - Change from Baseline**			
n	59	58	57
Mean (SD)	13.5 (50.6)	14.9 (40.8)	36.5 (58.5)
Median	9.9	16.1	20.0
Min, Max	-99.2, 220.5	-105.9, 114.2	-57.8, 228.7

qow: every other week; SD: standard deviation
