# Supplementary material for: Efficacy and safety of enzyme replacement therapy with BMN 110 (elosulfase alfa) for Morquio A syndrome (mucopolysaccharidosis IVA): a phase 3 randomised placebo-controlled study
Source: J Inherit Metab Dis. 2014 May 9;37(6):979–90. doi: 10.1007/s10545-014-9715-6 (PMC4206772; doi:10.1007/s10545-014-9715-6)
Supplement: Supplementary file 5 — (PDF 29 kb) [file 10545_2014_9715_MOESM5_ESM.pdf]

Supportive online material 5: ANCOVA of 6-minute walk test change from baseline to week 24: summary of subgroup by treatment interaction analysis. Intent to treat population

	Estimate	95% CI	*P*-value
**Baseline 6MWT category**			
Elosulfase alfa 2.0 mg/kg/qow vs placebo (6MWT ≤ 200 meters)	5.03	(-23.97,34.04)	
Elosulfase alfa 2.0 mg/kg/qow vs placebo (6MWT > 200 meters)	-2.19	(-25.79,21.41)	
Elosulfase alfa 2.0 mg/kg/qow vs placebo (6MWT > 200 meters - 6MWT≤ 200 meters)	-7.22	(-44.60,30.16)	0.7034
Elosulfase alfa 2.0 mg/kg/week vs placebo (6MWT ≤ 200 meters)	40.38	(11.00,69.76)	
Elosulfase alfa 2.0 mg/kg/week vs placebo (6MWT > 200 meters)	10.84	(-12.76,34.44)	
Elosulfase alfa 2.0 mg/kg/week vs placebo (6MWT > 200 meters - 6MWT≤ 200 meters)	-29.54	(-67.18,8.10)	0.1232
**Age group at baseline**			
Elosulfase alfa 2.0 mg/kg/qow vs placebo (Age 5-11)	-11.67	(-37.19,13.85)	
Elosulfase alfa 2.0 mg/kg/qow vs placebo (Age 12-18)	23.77	(-12.01,59.56)	
Elosulfase alfa 2.0 mg/kg/qow vs placebo (Age ≥ 19)	2.34	(-36.83,41.50)	
Elosulfase alfa 2.0 mg/kg/qow vs placebo (Age 12-18 - Age 5-11)	35.45	(-8.50,79.39)	0.1132
Elosulfase alfa 2.0 mg/kg/qow vs placebo (Age ≥ 19 - Age 5-11)	14.01	(-32.74,60.75)	0.5549
Elosulfase alfa 2.0 mg/kg/week vs placebo (Age 5-11)	13.76	(-11.54,39.07)	
Elosulfase alfa 2.0 mg/kg/week vs placebo (Age 12-18)	48.22	(12.44,84.01)	
Elosulfase alfa 2.0 mg/kg/week vs placebo (Age ≥ 19)	10.43	(-30.94,51.80)	
Elosulfase alfa 2.0 mg/kg/week vs placebo (Age 12-18 - Age 5-11)	34.46	(-9.36,78.29)	0.1224
Elosulfase alfa 2.0 mg/kg/week vs placebo (Age ≥ 19 - Age 5-11)	-3.34	(-51.83,45.15)	0.8921

	Estimate	95% CI	*P*-value
**Sex**			
Elosulfase alfa 2.0 mg/kg/qow vs mlacebo (Male)	6.81	(-19.15,32.76)	
Elosulfase alfa 2.0 mg/kg/qow vs placebo (Female)	-5.42	(-32.22,21.39)	
Elosulfase alfa 2.0 mg/kg/qow vs placebo (Female - Male)	-12.22	(-49.62,25.18)	0.5197
Elosulfase alfa 2.0 mg/kg/week vs placebo (Male)	27.27	(-0.34,54.89)	
Elosulfase alfa 2.0 mg/kg/week vs placebo (Female)	18.39	(-6.75,43.52)	
Elosulfase alfa 2.0 mg/kg/week vs placebo (Female - Male)	-8.89	(-46.21,28.43)	0.6388
**Race**			
Elosulfase alfa 2.0 mg/kg/qow vs placebo (White)	-5.94	(-28.28,16.40)	
Elosulfase alfa 2.0 mg/kg/qow vs placebo (Non-White)	14.11	(-18.39,46.61)	
Elosulfase alfa 2.0 mg/kg/qow vs placebo (Non-White - White)	20.05	(-19.38,59.48)	0.3168
Elosulfase alfa 2.0 mg/kg/week vs placebo (White)	31.45	(9.30,53.61)	
Elosulfase alfa 2.0 mg/kg/week vs placebo (Non-White)	11.23	(-21.91,44.37)	
Elosulfase alfa 2.0 mg/kg/week vs placebo (Non-White - White)	20.22	(-60.06,19.61)	0.3176
**Region**			
Elosulfase alfa 2.0 mg/kg/qow vs placebo (North America)	8.97	(-26.77,44.72)	
Elosulfase alfa 2.0 mg/kg/qow vs placebo (Europe)	-7.27	(-36.59,22.05)	
Elosulfase alfa 2.0 mg/kg/qow vs placebo (Other)	3.60	(-29.16,36.36)	
Elosulfase alfa 2.0 mg/kg/qow vs placebo (Europe - North America)	-16.24	(-62.96,30.47)	0.4933
Elosulfase alfa 2.0 mg/kg/qow vs placebo (Other - North America)	-5.37	(-53.88,43.13)	0.8271
Elosulfase alfa 2.0 mg/kg/week vs placebo (North America)	42.63	(5.88,79.37)	
Elosulfase alfa 2.0 mg/kg/week vs placebo (Europe)	23.51	(-4.59,51.61)	
Elosulfase alfa 2.0 mg/kg/week vs placebo (Other)	4.67	(-29.64,38.98)	
Elosulfase alfa 2.0 mg/kg/week vs placebo (Europe - North America)	-19.11	(-66.09,27.86)	0.4228
Elosulfase alfa 2.0 mg/kg/week vs placebo (Other - North America)	-37.95	(-88.40,12.50)	0.1393
