# Supplementary material for: Efficacy and safety of enzyme replacement therapy with BMN 110 (elosulfase alfa) for Morquio A syndrome (mucopolysaccharidosis IVA): a phase 3 randomised placebo-controlled study
Source: J Inherit Metab Dis. 2014 May 9;37(6):979–90. doi: 10.1007/s10545-014-9715-6 (PMC4206772; doi:10.1007/s10545-014-9715-6)

Supportive online matrial 6: Mean change from baseline over time in stairs/min climbed in a 3-minute stair climb test (3MSCT) (model-based repeated measures ANCOVA).

Error bars represent 95% CI of least squared mean change from baseline.

**P*-value vs. placebo

qow; every other week

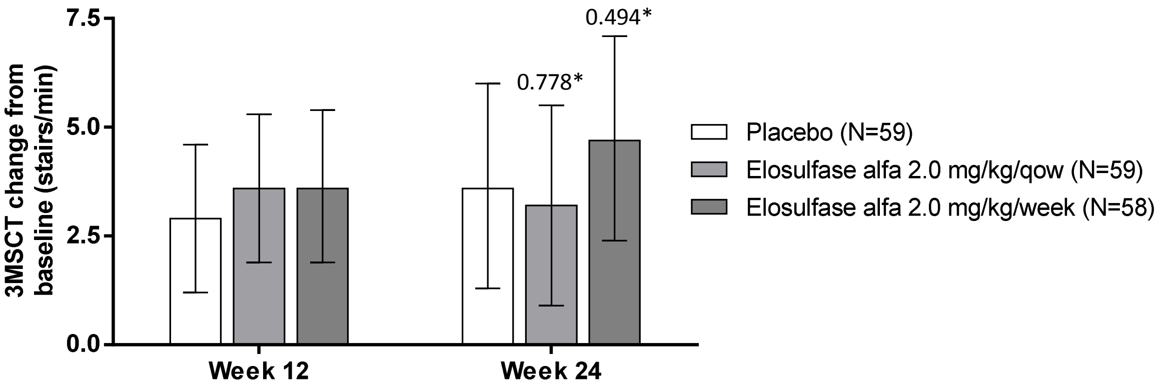

Supplement: Supplementary file 6 — (PDF 21 kb) [file 10545_2014_9715_MOESM6_ESM.pdf]
