# Supplementary material for: Efficacy and safety of enzyme replacement therapy with BMN 110 (elosulfase alfa) for Morquio A syndrome (mucopolysaccharidosis IVA): a phase 3 randomised placebo-controlled study
Source: J Inherit Metab Dis. 2014 May 9;37(6):979–90. doi: 10.1007/s10545-014-9715-6 (PMC4206772; doi:10.1007/s10545-014-9715-6)
Supplement: Supplementary file 7 — (PDF 19 kb) [file 10545_2014_9715_MOESM7_ESM.pdf]

Supportive online material 7: The effect of elosulfase alfa on primary, secondary, and tertiary efficacy endpoints (intent-to-treat population).

Modelled treatment effect, defined as (change from baseline to week 24, elosulfase alfa) – (change from baseline to week 24, Placebo)

**Modeled treatment effect**[a]	**2.0 mg/kg/qow vs. Placebo (N = 59 vs. 59)**	**2.0 mg/kg/week vs. Placebo (N = 58 vs. 59)**
**Primary outcome**		
**6MWT distance** (meters change from BL)		
LS Mean difference (95% CI)	0.5 (-17.8, 18.9)	22.5 (4.0, 40.9)
P-value[b]	*0.954*	*0.017*
**Secondary outcome**		
**3MSCT** (stairs/min change from BL)		
LS Mean difference (95% CI)	-0.5 (-3.7, 2.8)	1.1 (-2.1, 4.4)
P-value[c]	*0.778*	*0.494*
**Normalised urine KS** (% change from BL)		
LS Mean difference (95% CI)	-30.2 (-38.5, -22.0)	-40.7 (-49.0, -32.4)
P-value[d]	*<0.001*	*<0.001*
**Tertiary outcome**		
**MVV** (% change from BL)		
LS Mean difference (95% CI)	3.4 (-9.9, 16.6)	10.3 (-1.8, 22.4)
P-value[e]	*0.611*	*0.094*
**6MWD/3MSC/MVV** Composite z-score (change from BL)		
LS Mean difference (95% C)	0.0 (-0.1, 0.2)	0.1 (-0.0, 0.3)
P-value[f]	*0.882*	*0.053*
**FVC** (% change from BL)		
LS Mean difference (95% CI)	3.0 (-2.4, 8.3)	3.3 (-3.1, 9.6)
P-value[g]	*0.275*	*0.304*
**FEV_1** (% change from BL)		
LS Mean difference (95% CI)	0.2 (-7.4, 7.9)	1.8 (-5.5, 9.2)
P-value[h]	*0.955*	*0.613*
**MPS HAQ**		
**Self care domain**[m] **score** (change from BL)		
LS Mean difference (95% CI)	-0.1 (-0.5, 0.3)	0.1 (-0.3, 0.5)
P-value[i]	*0.724*	*0.737*
**Caregiver assistance domain**[m] **score** (change from BL)		
LS Mean difference (95% CI)	-0.4 (-2.4, 1.6)	-0.9 (-2.8, 1.1)
P-value[j]	*0.679*	*0.399*
**Mobility domain**[n] **score** (change from BL)		
LS Mean difference (95% CI)	-0.3 (-0.8, 0.2)	-0.3 (-0.8, 0.3)
P-value[k]	*0.261*	*0.336*
**Anthropometric measures**		
**Standing height z-score**[o] (change from BL)		
LS Mean difference (95%CI)	0.1 (-0.1, 0.3)	0.1 (-0.0, 0.3)
P-valuk	*0.222*	*0.115*
**Growth rate z-score**[o] (change from BL)		
LS Mean difference (95% CI)	0.4 (-0.1, 0.9)	0.4 (-0.1, 0.9)
P-value[m]	*0.138*	*0.103*

3MSCT: 3-minute stair climb test; 6MWT: 6-minute walk test; BL: baseline; CI: confidence interval; FVC: forced vital capacity; HAQ: health assessment questionnaire; KS: keratan sulfate; LS Mean: least

squares mean; MPS: mucopolysaccharidosis; MVV: maximum voluntary ventilation; qow: every other week

[a]Treatment effect defined as (change from baseline to week 24, elosulfase alfa) – (change from baseline to week 24, placebo)

[b]*P*-value determined by t-test from ANCOVA model (primary endpoint analysis), adjusted for baseline covariates: age group and 6-minute walk test (6MWT) category

[c]*P*-value determined by t-test ANCOVA model adjusted for baseline covariates: age group, 6MWT category, and continuous 3-minute stair climb test

[d]*P*-value determined by t-test ANCOVA model adjusted for baseline covariates: age group, 6MWT category, and normalized urine KS

[e-m] *P*-value determined by t-test from ANCOVA model adjusted for baseline covariates: age group, 6MWT category, and respectively MVV[e], composite score[f], FVC[g], FEV_1[h], MPS HAQ Self care domain score[i], MPS HAQ Caregiver assistance domain score[j], MPS HAQ Mobility domain score[k], standing height[l], and growth rate[m]

[n]MPS HAQ domain scores range from 0 to 20; decreases in domain scores (negative values) indicate improvement

[o]The normalised standing height (scaled as a z score) computed using CDC normals was explored in males ≤18 years and females ≤15 years; the combined historical and on study normalized standard height z scores were used to compute growth rate change (scaled as z-score)

Tertiary variables that were analysed differently from those included in the table are discussed in the text
